# Spirituality is Associated with Immune Parameters and Disease Activity in Primary Sjögren’s Syndrome: A Cross-Sectional Study

**DOI:** 10.1192/j.eurpsy.2024.1426

**Published:** 2024-08-27

**Authors:** L. Módis, Z. Aradi, F. I. Horváth, P. Pikó, A. Szántó, A. Bugán

**Affiliations:** ^1^Faculty of Medicine, Department of Behavioural Sciences, University of Debrecen, Debrecen; ^2^Sántha Kálmán Member Hospital, Szabolcs Szatmár Bereg County Teaching Hospital, Nagykálló; ^3^Faculty of Medicine, Department of Internal Medicine, Division of Clinical Immunology; ^4^Faculty of Medicine, Department of Public Health and Epidemiology, University of Debrecen, Debrecen; ^5^National Laboratory for Health Security, Center for Epidemiology and Surveillance, Semmelweis University, Budapest, Hungary

## Abstract

**Introduction:**

The role of spirituality in health and disease is a complex and emerging area of research. Incorporating spirituality into the bio-psycho-social model of health and disease leading to the bio-psycho-social-spiritual model provides a more comprehensive framework. In this context, chronic disorders like primary Sjögren’s syndrome (pSS) are of interest due to their intricate interactions between biological, psychological, and spiritual factors.

**Objectives:**

To study possible relationships between spirituality, immune parameters, and disease activity in pSS patients.

**Methods:**

Patient recruitment for the study took place at the Autoimmune Sjögren specialty clinic, University of Debrecen, resulting in 112 patients. Assessing spirituality of the patients happened through 4 direct questions and the Sprituality Transcendence Scale (24 items). Besides, clinical data of the patients were involved in the study including blood cell counts, rheumatoid factor, immunoglobulin G, Sjögren-specific autoantibodies and disease activity scores (semi-objective and patient reported,). The statistical analysis was conducted applying group comparisons between spiritual and non-spiritual groups, and linear and logistic regression analyses adjusted for sex, age, disease duration, settlement type, education, living in partnership and smoking. Out of the 112 patients 4 gave incomplete response, and therefore got excluded from the analysis, resulting in a total sample size of 108.

**Results:**

Semi-objective disease activity score (ESSDAI) and perceived vaginal dryness was significantly lower in the non-spiritual group. Spirituality was proven as a significant predictor of anti-SSB autoantibody serum activity and ESSDAI, while engaging in prayer/meditation and its duration predicted significantly anti-SSA autoantibody serum activity, perceived skin and tracheal dryness. Concerning logistic regression analysis, we found that an increase of one unit in spirituality reduces the probability with 81.6% of having a detectable, semi-objective disease activity at all. Significant associations were found between the duration of prayer/meditation and both semi-objective and patient reported disease activity scores and autoantibody anti-SSB with an inverse ratio based on logistic regression model.

**Conclusions:**

Spirituality is associated with immune parameters and disease activity in pSS. Patients with spiritual attitude are less likely to have increased disease activity. Besides being spiritual, engagement in individual spiritual activities, such as prayer/meditation has beneficial disease modifying effect. These changes are supposedly due to psychoneuroimmunological pathways. In addition to the biologically measurable variables, the alleviation and aggravation of perceived symptoms (e.g. dryness) are important outcomes of spiritual engagement and practice.

**Disclosure of Interest:**

None Declared

